# Artificial Intelligence and Machine Learning in Hospital Quality Management, Patient Safety, and Accreditation Readiness: A Systematic Review and Narrative Synthesis

**DOI:** 10.7759/cureus.109393

**Published:** 2026-05-21

**Authors:** Nitin Marathe, Manish Ranjan, Madhusudan P Singh, Pragati Trigunait, Avinash Prakash, Nitin Kaushal, Amit Pawar, Girish Singh Kshatriya, Rounak Dubey

**Affiliations:** 1 Hospital Administration, All India Institute of Medical Sciences, Nagpur, Nagpur, IND; 2 Microbiology, All India Institute of Medical Sciences, Jammu, Jammu, IND; 3 Pharmacology and Therapeutics, All India Institute of Medical Sciences, Deoghar, Deoghar, IND; 4 Obstetrics and Gynaecology, All India Institute of Medical Sciences, Nagpur, Nagpur, IND; 5 Anaesthesiology, All India Institute of Medical Sciences, Nagpur, Nagpur, IND; 6 Community Medicine and Public Health, Postgraduate Institute of Medical Education and Research, Chandigarh, IND; 7 Transfusion Medicine, Military Hospital Doda, Jammu, IND; 8 Transfusion Medicine, All India Institute of Medical Sciences, Deoghar, Deoghar, IND; 9 Transfusion Medicine, All India Institute of Medical Sciences, Nagpur, Nagpur, IND

**Keywords:** accreditation, artificial intelligence, deep learning, healthcare governance, hospital quality management, machine learning, natural language processing, patient safety, quality assurance

## Abstract

As healthcare systems become increasingly data-driven, artificial intelligence (AI) and machine learning (ML) are being explored as tools to support quality management, patient safety, and accreditation. This systematic review synthesizes the available evidence across these domains through a structured literature search of major biomedical databases, encompassing studies published over the past decade and narratively synthesized to capture implementation context alongside technical performance. Three electronic databases were searched, and 23 studies were included. The available empirical evidence suggests that these technologies are most useful for repetitive, operationally well-defined, and digitally captured tasks, while local validation, acceptance testing, and continuous monitoring consistently emerge as prerequisites for safe institutional deployment. Governance capacity and organizational readiness are identified as equally determinative of outcomes as algorithmic performance itself. Collectively, the evidence supports positioning AI and ML as augmentative instruments within human-led quality governance structures, rather than as autonomous operational systems. Evidence was heterogeneous, with most empirical studies being single-center, retrospective, or implementation-focused. The risk-of-bias assessment indicated limited high-quality comparative evidence, limiting the strength of the pooled conclusions.

## Introduction and background

Hospitals are increasingly becoming digitized and generating substantial clinical, operational, and administrative data; however, the extent of digitization remains uneven, and data fragmentation persists in many under-resourced settings. Electronic health records, incident-reporting systems, imaging platforms, maintenance logs, and accreditation-related dashboards all contribute to an expanding quality-data infrastructure. Against this backdrop, artificial intelligence (AI) and machine learning (ML) have emerged as candidate tools for transforming complex data streams into operationally relevant quality intelligence [1,2]. 

The potential of AI in hospital systems extends well beyond diagnosis and clinical decision support. It now encompasses safety surveillance, audit support, workflow standardization, quality-control review, post-market device monitoring, and accreditation readiness, defined as institutional preparedness to demonstrate compliance with external quality and safety standards. AI can support routine quality work by flagging delayed critical-result communication, identifying recurring safety incidents, summarizing audit data, and monitoring accreditation-related documentation. At the same time, important concerns remain after these tools are deployed in routine care. These include limited generalizability, bias, lack of transparency, and unclear accountability. Another concern is model drift, where model performance may decline over time as patient populations, workflows, or data systems change. Hospital implementation studies and accreditation-oriented frameworks have repeatedly cautioned that strong developmental performance does not, on its own, guarantee safe local use [3,4].

This issue is particularly important in hospital quality management because these systems influence institutional oversight, safety surveillance, and accreditation preparedness. It encompasses compliance monitoring, adverse-event review, documentation quality, operational reliability, and accreditation-linked quality assurance. These functions require more than good technical performance. They also need strong validation, traceability, and AI governance. AI governance refers to the institutional oversight, monitoring, and accountability processes needed for safe AI deployment. Existing work on AI quality management and healthcare regulation increasingly emphasizes that hospitals must actively oversee AI systems to ensure their safe use [5-7].

The present review was designed to address three objectives: first, to evaluate the reported effectiveness and performance of AI and ML interventions in hospital quality management, patient safety, and accreditation-related outcomes; second, to assess validation, implementation, and governance considerations relevant to safe institutional deployment; and third, to identify gaps in generalizability, and implementation that bear directly on hospital quality and patient safety functions.

## Review

Methods

Review Design and Reporting

This systematic review adhered to the PRISMA 2020 statement [8], with the abstract structured according to the PRISMA 2020 for Abstracts checklist and search methods documented following PRISMA-S [9]. The protocol was prospectively registered with PROSPERO on 25 January 2026 (https://www.crd.york.ac.uk/PROSPERO/view/CRD420261292821).

Eligibility Criteria

Studies were assessed against a two-tier eligibility framework designed to capture both empirical hospital-based evidence and the cross-cutting governance literature that shapes hospital AI quality practice. This approach was adopted in recognition of the field's emerging nature, where institution-specific empirical evidence remains limited and is substantially informed by external regulatory, safety-framework, and governance.

Under the first tier, studies were eligible if they were conducted in hospitals or hospital-based healthcare systems, including tertiary hospitals, secondary hospitals, academic medical centers, and public or private institutions, provided they addressed hospital quality teams, patient safety programs, accreditation functions, imaging quality programs, or institution-wide quality systems. Under the second tier, studies were eligible if they addressed AI governance frameworks, regulatory standards, safety guardrails, certification systems, or quality-assurance methodologies that demonstrated direct translational relevance to hospital quality assurance, accreditation, or patient safety functions.

Eligible interventions included AI and ML applications, comprising ML models, deep learning, natural language processing (NLP), computer vision, expert systems, and LLMs, when applied to hospital quality management, patient safety, audit, compliance, or accreditation-related processes. Primary outcomes included adverse-event detection, quality-indicator reporting, audit efficiency, compliance rates, and accreditation-related performance metrics. Secondary outcomes encompassed model performance metrics (sensitivity, specificity, accuracy, area under the curve (AUC)), process outcomes, such as workload reduction, implementation feasibility, and governance or safety considerations.

Information Sources and Search Strategy

PubMed, Embase, and Scopus were searched for English-language studies published from 01 January 2016 to 01 January 2026. The search strategy was organized around four conceptual domains: (1) AI and ML; (2) hospital settings; (3) quality management and patient safety; and (4) accreditation, audit, or compliance. Terms within each domain were combined using OR operators, and domains were linked with AND. The complete search strings for all databases are provided in Appendix 1.

Article types included are observational studies, interventional and implementation studies, predictive model development and validation studies, mixed-methods studies, and systematic reviews (for evidence mapping).

Article types excluded are editorials, opinion pieces, commentaries, conference abstracts, preprints, and non-peer-reviewed articles.

Screening and Study Selection

All retrieved references were imported into Rayyan (Rayyan Systems Inc., Cambridge, MA), a web-based collaborative platform designed to support blinded title and abstract screening in systematic reviews through duplicate detection, label-based decisions, and inter-reviewer conflict [10]. Two reviewers (PT, RD) independently screened titles and abstracts, followed by a full-text review of potentially eligible articles. Disagreements were resolved by adjudication with a third reviewer (NM). 

Data Extraction and Appraisal

Data extraction was performed independently by two reviewers (NK, AP) using a standardized form capturing the following: study design, country and setting, AI modality and specific algorithm, hospital quality domain addressed, comparator where applicable, primary and secondary outcomes, key performance metrics, validation approach, implementation characteristics, and governance or safety considerations. Discrepancies were resolved by consensus or adjudication by the third reviewer (NM). Governance-oriented, regulatory, and conceptual papers were appraised descriptively for scope and institutional relevance using four structured criteria: clarity of scope, evidence basis, transferability to hospital quality management, and overall quality rating.

For prediction model studies, risk of bias was assessed using PROBAST (Prediction model Risk Of Bias Assessment Tool), evaluating four domains: participants, predictors, outcome, and analysis. For observational and implementation studies, risk of bias was assessed using ROBINS-I (Risk of Bias In Non-randomized Studies of Interventions) across seven domains. The included systematic review was appraised using AMSTAR-2 (A MeaSurement Tool to Assess systematic Reviews). Assessments were performed independently by two reviewers, with disagreements resolved by adjudication by the third reviewer.

Synthesis Strategy

Given the heterogeneity of interventions, designs, and outcome measures across included studies, synthesis was primarily narrative and organized thematically by hospital quality domain. Selected studies reporting clearly extractable technical performance metrics were summarized descriptively to provide quantitative context for the thematic synthesis. No formal pooled meta-analytic estimate was calculated, owing to the heterogeneity of endpoints and AI modalities across the evidence base.

Results

Study Selection

**Figure 1:**
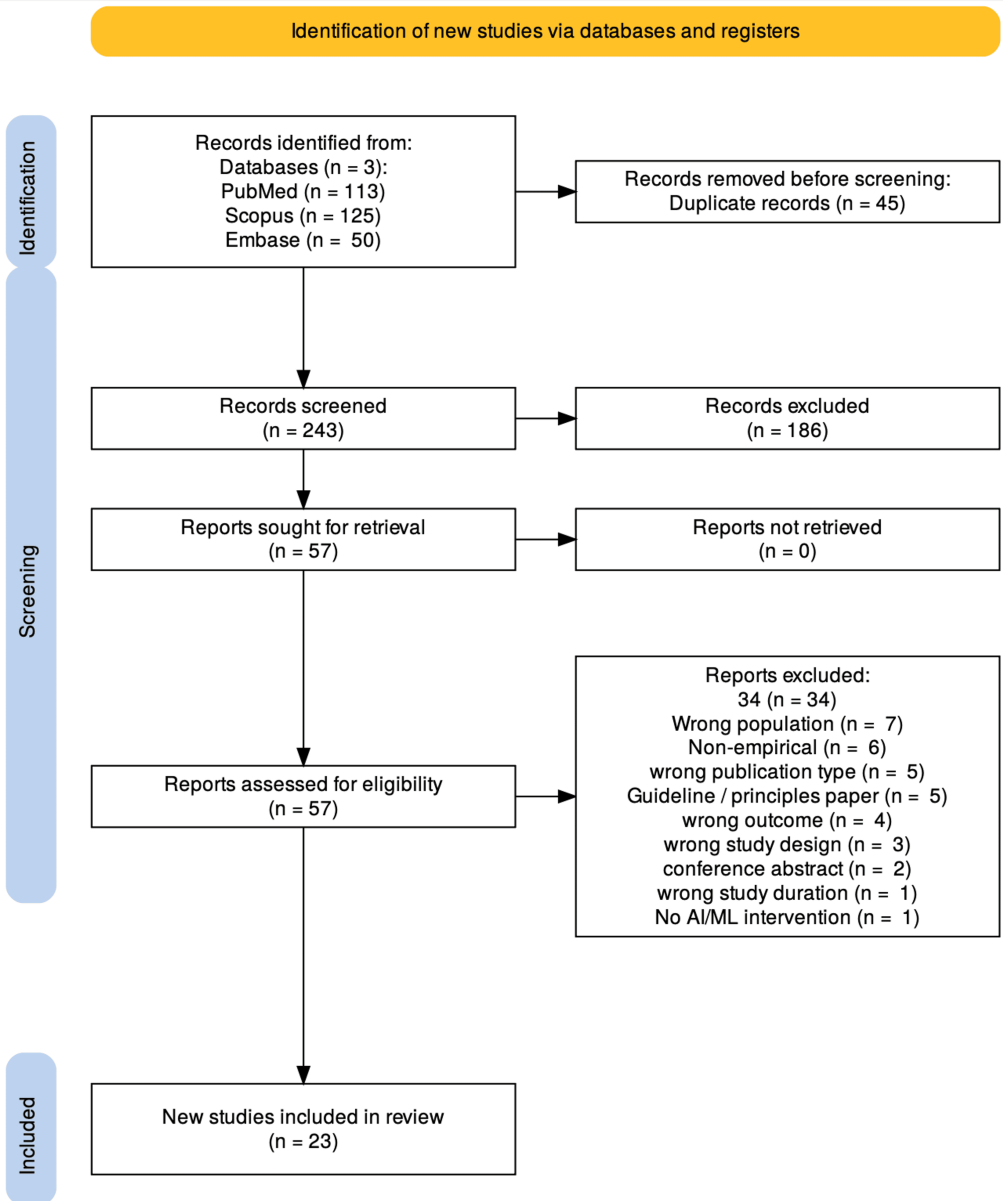
PRISMA flow diagram illustrating the study selection process

Database searches identified 288 records. After removal of 45 duplicates, 243 unique records underwent title and abstract screening by two reviewers (PT, RD). Fifty-seven reports advanced to full-text retrieval, of which 23 articles met all eligibility criteria and were included in the final review. Screening was performed in duplicate, in a blinded manner, on Rayyan, with conflicts adjudicated by a third reviewer (NM). The PRISMA flow diagram is presented in Figure 1.

Characteristics of Included Studies

The 23 included studies represented a broad evidence base spanning empirical investigations, quality-improvement studies, implementation studies, systematic reviews, and governance or regulatory analyses. Studies were published between 2019 and 2025. The evidence addressed several recurring domains of hospital quality management: surveillance and audit of safety-critical events; documentation quality and workflow standardization; accreditation-linked imaging quality assurance; predictive maintenance and operational reliability; patient-perceived service quality; and institutional governance, readiness, and lifecycle oversight. The characteristics of the included studies are presented in Table 1. Studies are organized into four categories: hospital-based empirical and implementation studies (S. No. 1-10), the included systematic review (S. No. 11), conceptual/regulatory/governance studies with direct relevance to hospital quality management (S. No. 12-19), and conceptual/regulatory/governance studies with indirect relevance retained under the second-tier governance eligibility criterion (S. No. 20-23, marked *). Section headers and subtle row shading reflect these groupings.

**Table 1:** Characteristics of included studies (n = 23) ANN, artificial neural network; CNN, convolutional neural network; CPD, continuing professional development; EHR, electronic health record; IoT, Internet of Things; LLM, large language model; ML, machine learning; NLP, natural language processing; QA, quality assurance; QC, quality control; QI, quality improvement; QM, quality management; RF, random forest; SVM, support vector machine; XR, extended reality Evidence type categories: Empirical (Hospital) = primary data collected in a hospital quality, safety, or accreditation setting; Empirical (Operational / Technical) = primary technical or operational data with translational relevance to hospital workflows; Implementation / QI = prospective quality-improvement or implementation evaluation; Systematic Review = review-level evidence synthesis; Conceptual / Regulatory / Governance = framework, regulatory, or governance papers without primary hospital outcome data. * Studies marked with an asterisk [23-26] were retained under the second-tier governance eligibility criterion and provide contextual rather than direct hospital-based evidence; their findings should not be weighted equally with hospital-based empirical studies.

S. No.	First Author (Year)	Country	Journal	Study Design	AI Modality	Hospital Quality Domain	Evidence Type	Key Findings / Contribution
Empirical and Implementation Studies (Hospital-Based)								
1	Heilbrun et al. (2019) [11]	USA	J Am Coll Radiol	Retrospective feasibility study	Rule-based NLP	Critical findings auditing (radiology)	Empirical (Hospital)	NLP identified 69/75 critical findings; sensitivity 0.92, specificity 0.99; feasible for communication audit.
2	Kobritz et al. (2023) [12]	USA	J Surg Res	Comparative study (3 centers)	NLP	Morbidity and mortality review	Empirical (Hospital)	NLP identified 670 complications vs 611 by conventional database; <30% overlap; AI broadens but does not replace clinician review.
3	Small et al. (2025) [13]	USA	JAMA Netw Open	Quality-improvement study	LLM (EHR-embedded)	Documentation support	Implementation / QI	LLM summaries needed fewer edits and were more complete but more prone to confabulation; mandates clinician verification.
4	Rahim et al. (2021) [14]	Malaysia	Int J Environ Res Public Health	ML-based text analysis	Supervised ML (sentiment)	Patient experience monitoring	Empirical (Hospital)	72.1% positive, 27.9% negative sentiment in Facebook reviews; most SERVQUAL dimensions associated with positive sentiment. Hypothesis-generating only.
5	Dattu et al. (2025) [15]	Malaysia	Int J Health Care Qual Assur	Predictive modelling (6 hospitals)	RF, ANN, SVM	Predictive maintenance (critical devices)	Empirical (Operational / Technical)	RF/ANN reported accuracy 1.00, F1 0.90; SVM recall 0.94; estimated 12–15% improvement in spare-parts availability. Reported perfect accuracy is methodologically implausible without external validation; downweighted in synthesis.
6	Yun et al. (2025) [16]	South Korea	PLoS One	Retrospective model validation	Deep learning (CNN)	Mammography phantom QA / accreditation	Empirical (Hospital)	Accuracy: fibers 87.84%, specks 93.43%, masses 86.63%; AUC > 0.97 for lesion presence. Supports accreditation-linked imaging QC.
7	Sunami et al. (2024) [17]	Japan	JAMA Oncol	Prospective quality improvement	AI treatment recommendation system	Multidisciplinary review standardization	Implementation / QI	Tumor-board concordance rose from 58.7% to 67.9% after the learning program; AI achieved 88.0% concordance. Standardizes multidisciplinary review.
8	Hundur et al. (2025) [18]	Bosnia and Herzegovina	Technol Health Care	ECG device case study	AI-assisted post-market surveillance	Post-market device surveillance	Empirical (Operational/Technical)	Demonstrated feasibility of ongoing surveillance for ECG devices; supports continuous monitoring after deployment.
9	Lundström et al. (2023) [3]	Sweden	J Digit Imaging	Landscape mapping study	Quality-assurance frameworks	AI QA in diagnostic imaging	Empirical (Hospital)	Mapped QA approaches across care providers; found vendor validation insufficient for local safety.
10	Ranjbar et al. (2024) [1]	Norway	Risk Manag Healthc Policy	Gap analysis (ISO 42001)	AI management systems	Governance and readiness	Empirical (Hospital)	Identified readiness deficits in hospital AI management systems; technical debt and fragmented data infrastructure as primary barriers.
Systematic Review								
11	Waldock et al. (2024) [2]	UK	J Med Internet Res	Systematic review and meta-analysis	Multiple AI types	AI accuracy benchmarking	Systematic Review	AI showed promising capability on healthcare assessments; highlights need for local validation and context-specific evaluation.
Conceptual / Regulatory / Governance Studies - Direct Relevance								
12	Larson et al. (2025) [4]	USA	J Am Coll Radiol	Roadmap/framework paper	AI accreditation systems	Accreditation for radiology AI	Conceptual / Regulatory / Governance	Described ARCH-AI; local acceptance testing, monitoring, governance, and physician oversight required.
13	Zanca et al. (2022) [5]	Multinational	Semin Radiat Oncol	Regulatory review	AI medical software regulation	Regulatory and lifecycle oversight	Conceptual / Regulatory / Governance	Analyzed EU and US regulatory frameworks; emphasized procurement oversight, post-market surveillance, and lifecycle clarity.
14	Mercolli et al. (2024) [6]	Switzerland	Z Med Phys	Conceptual framework paper	AI quality management	Quality management of AI systems	Conceptual / Regulatory / Governance	Proposed risk-stratified management of AI systems aligned with clinical risk and workflow context.
15	Welzel et al. (2023) [7]	Germany	J Med Internet Res	Systems-level analysis	Automated assessment tools	Holistic digitization/system assessment	Conceptual / Regulatory / Governance	Advocated integrated automated system-level tools for holistic care digitization; emphasized human-centered design.
16	Hakim et al. (2025) [19]	USA	Sci Rep	Conceptual / guardrails paper	LLM guardrails	Patient safety in LLM deployment	Conceptual / Regulatory / Governance	Proposed structured guardrails for LLMs in pharmacovigilance; highlighted hallucination risk in safety-critical settings.
17	Nehme et al. (2024) [20]	Switzerland	Swiss Med Wkly	Regulatory / governance review	Medical chatbot	Chatbot certification / regulation	Conceptual / Regulatory / Governance	Showed that the intended-use specification determines regulatory classification and defines QM obligations for chatbots.
18	Zinchenko et al. (2021) [21]	Russia	Kazan Med J	Regulatory / standardization review	AI governance standards	National AI regulation (Russia)	Conceptual / Regulatory / Governance	Described national AI standards encompassing clinical/technical trials, lifecycle and risk management, and dataset governance.
19	Newman-Griffis (2019) [22]	USA	BMC Public Health	Informatics framework paper	NLP for functional data	Functional data capture in health systems	Conceptual / Regulatory / Governance	Argued for NLP-supported extraction of activity/function information; addresses standardization of health information data.
Conceptual / Regulatory / Governance Studies - Indirect Relevance *								
20	McMahon (2025) [23] *	USA	J Contin Educ Health Prof	Narrative review	AI oversight systems	AI in CPD accreditation	Conceptual / Regulatory / Governance	Discussed risk-based audit and pattern recognition in accreditation; supports human adjudication. Indirect relevance to hospital accreditation.
21	Rowan (2024) [24] *	Ireland	Sci Total Environ	Narrative review	Digital twin / extended reality	Medical device and training ecosystem	Conceptual / Regulatory / Governance	Reviewed digital twin and XR applications relevant to lifecycle monitoring and safe device design. Indirect relevance to hospital QM.
22	Machal (2024) [25] *	Finland	Front Med Technol	Review paper	AI-powered autoinjectors	AI in medical device functionality	Conceptual / Regulatory / Governance	Examined risks/benefits of AI autoinjectors relevant to safety governance for AI-enabled devices. Device-focused; indirect relevance.
23	Ma (2022) [26] *	China	Comput Intell Neurosci	Technical development study	IoT + deep learning	Archives management / professional certification	Conceptual / Regulatory / Governance	Autonomous archive management system using deep learning; contextually relevant to institutional certification workflows. Indirect relevance.

**Figure 2:**
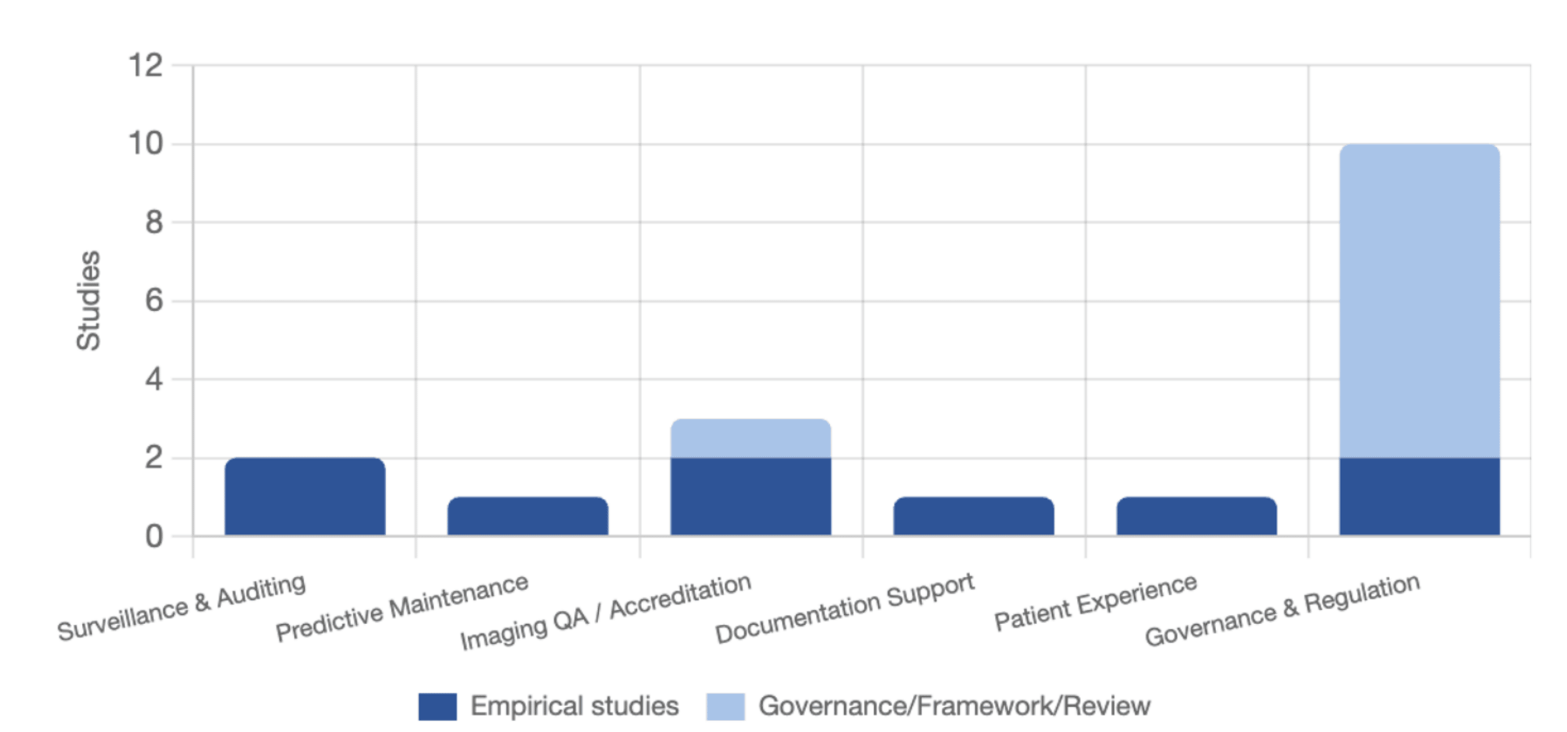
Distribution of included studies by primary hospital quality management domain

**Figure 3:**
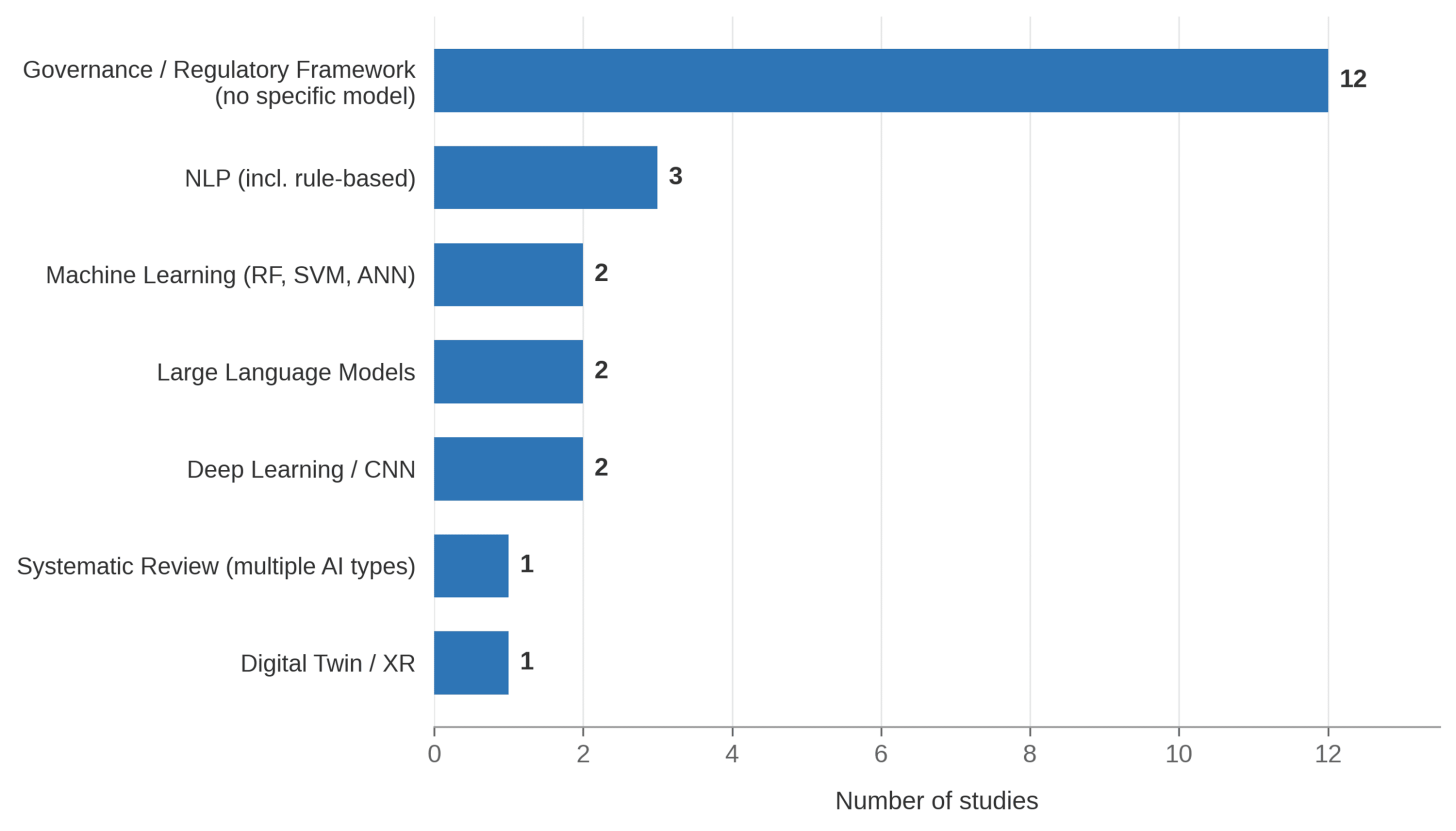
Distribution of included studies by AI modality ANN, artificial neural network; CNN, convolutional neural network; NLP, natural language processing; RF, random forest; SVM, support vector machine; XR, extended reality

The literature was heterogeneous in AI modality, study design, setting, and endpoint. Empirical studies were predominantly single-center or limited-network investigations. Several papers addressed institutional and regulatory frameworks for safe AI adoption rather than direct quality-improvement outcomes. The distribution of studies across hospital quality domains and AI modalities is shown in Figure 2 and Figure 3.

Risk of Bias Assessment

Formal methodological appraisal was conducted only for studies eligible for structured assessment using design-appropriate tools. Specifically, we applied ROBINS-I to eligible non-randomized studies, PROBAST to prediction model studies, and AMSTAR 2 to systematic reviews, based on study design and methodological applicability. In summary, of the three prediction model studies appraised using PROBAST, two were rated at low overall risk of bias (Heilbrun et al. [11]; Yun et al. [16]), and one at "high" overall risk (Dattu et al. [15]), primarily because a reported classification accuracy of 1.00 for random forest and artificial neural network models raised concern about potential overfitting, and external validation data were not provided (Appendix 2).

Of the four observational and implementation studies assessed with ROBINS-I, three were rated as having a moderate overall risk of bias, and one was rated as having a serious risk of bias. Key concerns included selection bias in the social-media review study, Rahim et al. [14], which drew on Facebook reviews as a convenience sample. The other three studies included limited generalizability from simulated case scenarios (Sunami et al. [17]), artificial time constraints in the quality-improvement design (Small et al. [13]), and the retrospective multi-center design of Kobritz et al. [12] introduced moderate confounding risk from case-mix differences across sites (Appendix 3).

The included systematic review (Waldock et al. [2]) was appraised using AMSTAR-2 and rated at low confidence overall, principally because the publication bias was not assessed and the impact of risk of bias on pooled estimates was not formally modelled. For the 14 governance-oriented, regulatory, and conceptual papers, descriptive appraisal found that most were rated high or moderate overall quality; the principal weakness across this group was reliance on expert opinion or regulatory interpretation rather than primary empirical data, which limits the strength of evidence that can be attributed to their conclusions (Appendix 4).

Taken together, the risk-of-bias profile of the included literature is consistent with the early-stage and heterogeneous nature of evidence in this domain. The two empirical studies rated at low overall bias (Heilbrun et al. [11] and Yun et al. [16]) provide the most methodologically direct evidence among included studies, although both remain domain-specific and may not generalize across hospital quality functions. Findings from the remaining empirical studies should be interpreted in light of their moderate bias ratings and the methodological limitations identified above. The governance and regulatory literature, while rated largely high or moderate quality, contributes evidence in terms of institutional and policy context rather than performance data, and should be weighted accordingly.

Thematic Synthesis

AI for surveillance, auditing, and adverse-event detection: One of the central use cases that emerged from the review was the deployment of NLP to support surveillance and quality auditing. Heilbrun and colleagues evaluated a rule-based NLP system designed to identify critical findings in chest radiology reports as part of a retrospective quality assurance audit [11]. Working with a test set of 666 reports, of which 75 contained critical findings, and 591 did not, the system correctly flagged 69 of the 75 critical reports, yielding a sensitivity of 0.92 and specificity of 0.99. The high specificity carries particular operational significance as it substantially reduces the false-positive burden on audit staff, thereby enabling reviewers to concentrate on genuinely missed communications.

Kobritz and colleagues undertook a comparative analysis of NLP-supported morbidity and mortality review across 7,774 surgical admissions at three academic medical centers [12]. Across all methods combined, 987 cases with complications were identified. The conventionally maintained prospective database captured 611 cases, while the NLP-supported method identified 670 cases. Strikingly, the two systems overlapped in fewer than one-third of identified cases; the conventional database more frequently captured major-harm complications, whereas the NLP-based approach tended to flag lower-severity events. This complementary pattern, rather than outright substitution, has direct implications for quality management: AI broadens surveillance breadth but does not remove the need for clinician-led review and severity classification in morbidity and mortality programs.

AI for documentation quality and workflow support: Small and colleagues evaluated an electronic health record (EHR)-embedded large language model for hospital-course summarization in a prospective quality-improvement study [13]. The LLM-generated summaries required fewer edits from residents and were rated as more complete by attending physicians compared with physician-authored summaries. That said, the model-generated summaries were also rated as more susceptible to confabulation. From the standpoint of hospital quality systems, this is a clinically meaningful observation: documentation quality directly affects handovers, discharge safety, auditability, and continuity of care. The evidence suggests that LLMs may alleviate documentation burden and enhance completeness, but only when embedded within robust human-review processes. Given the known risk of hallucination/confabulation, LLM-generated summaries should not be used without clinician verification, audit trails, and clear accountability for final documentation.

AI for patient-perceived service quality monitoring: Rahim and colleagues applied supervised ML to 1,852 Facebook reviews from 48 Malaysian public hospitals to extract service-quality signals [14]. Their sentiment classifier categorized 72.1% of reviews as positive and 27.9% as negative. When mapped to SERVQUAL domains, most service-quality dimensions, with the exception of tangible factors, were associated with positive sentiment after covariate adjustment. Although patient-generated social-media data are inherently selective, this work demonstrates that ML-based monitoring may furnish hospitals with more timely quality signals than traditional periodic patient-experience surveys and could complement formal accreditation-linked survey instruments. This evidence should be interpreted as hypothesis-generating only and not as a substitute for validated patient-experience instruments such as HCAHPS or equivalent national survey frameworks.

AI for operational quality and predictive maintenance: Dattu and colleagues examined the application of ML to spare-parts availability for critical medical devices, drawing on 2,800 maintenance records from six hospitals covering ten device categories [15]. Their hybrid framework integrated failure mode and effects analysis (FMEA)-derived risk priority numbers with supervised ML algorithms. The random forest and ANN models each achieved classification accuracy of 1.00 and an F1 score of 0.90, while the SVM model achieved a recall of 0.94. The reported perfect accuracy should be interpreted cautiously until externally validated, as such performance may reflect overfitting, data leakage, or dataset-specific classification patterns. These findings are reported here for completeness but have been downweighted in the overall evidence synthesis and should be considered preliminary until externally validated in independent maintenance datasets. 

AI for accreditation-linked quality assurance: Yun and colleagues developed and validated a deep learning model for automated mammography phantom image evaluation within South Korea’s national accreditation program [16]. After preprocessing, 5,813 phantom images were retained. The model achieved classification accuracy of 87.84% for fibers, 93.43% for specks, and 86.63% for masses, with AUC > 0.97 for lesion presence, and > 0.94 for abnormality detection scoring categories. These findings indicate that deep learning can support consistency and efficiency in accreditation-linked imaging quality-control workflows, reducing inter-reader variability and reviewer burden in national programs.

Sunami and colleagues examined concordance in treatment recommendations among molecular tumor boards and an AI system in a prospective quality-improvement design [17]. Using 50 simulated cases and 42 eligible participants, concordance among tumor boards rose from 58.7% before the learning program to 67.9% after. The AI system achieved 88.0% concordance in post-learning evaluations, exceeding that of tumor boards. While situated in precision oncology, this study is relevant to hospital quality management because it addresses standardization of multidisciplinary review processes, a recurring challenge in hospital quality programs.

AI governance, institutional readiness, and lifecycle oversight: Hakim and colleagues proposed guardrails for LLMs in pharmacovigilance and other safety-critical medical contexts, identifying uncertainty communication, structured constraints, and drug-event matching as essential safeguards against hallucination and error propagation [19].

Nehme and colleagues described certification and regulatory considerations for medical chatbots, showing that intended-use specification determines whether a chatbot is regulated as a medical device and thereby determines its quality-management obligations [20]. Zinchenko and colleagues reviewed Russian national standardization efforts for healthcare AI, emphasizing that formal standards for lifecycle management, clinical and technical trials, risk management, and dataset governance are prerequisites for regulated AI use [21]. McMahon described the role of AI in continuing professional development accreditation oversight, illustrating AI support for risk-based auditing and pattern recognition while maintaining the primacy of human adjudication [23].

At the systems level, Rowan reviewed digital twin and extended reality applications in medical device ecosystems, highlighting their relevance to training, lifecycle monitoring, and safe design of complex hospital systems [24]. Newman-Griffis and colleagues argued for improved capture and NLP-supported standardization of functional information across health systems, an informatics challenge directly relevant to quality-data completeness [22]. Machal reviewed the risks and benefits of AI-powered autoinjectors with relevance to safety governance frameworks for AI-enabled devices [25]. Ma and colleagues described an autonomous archive management system using IoT and deep learning for professional certification, providing contextual evidence on AI in institutional certification workflows [26].

Governance and readiness: cross-cutting evidence: A hospital-focused gap analysis by Ranjbar and colleagues using ISO 42001 as a reference framework identified significant readiness deficits among healthcare organizations seeking to deploy AI at scale [1]. Key gaps included technical debt in legacy systems, fragmented data infrastructure, and insufficient AI-specific management-system maturity. This study reframes implementation risk as a governance issue rather than a purely technical one, a finding with direct implications for hospital quality leaders. Its application to healthcare AI is, however, still nascent, and published empirical evidence on hospital-level adoption and outcomes remains sparse. The systematic review by Waldock and colleagues similarly emphasized the need for context-specific evaluation rather than reliance on aggregate performance benchmarks [2].

Summary of Studies Reporting Extractable Performance Data

Although this review did not undertake formal meta-analytic pooling, three studies provided sufficiently extractable technical performance data to support a descriptive quantitative summary (Table 2). These three studies represent the most methodologically bounded and outcome-specific evidence currently available for AI in hospital quality applications. Evidence from the other studies has been summarized in Table 3. 

**Table 2:** Descriptive summary of studies reporting extractable technical performance data ANN, artificial neural network; CNN, convolutional neural network; NLP, natural language processing; QA, quality assurance; QC, quality control; RF, random forest; SVM, support vector machine

Study	AI Modality	Hospital Quality Domain	Dataset	Performance Metrics	Operational Implication
Heilbrun et al. [11]	Rule-based NLP	Critical findings auditing (chest radiology)	666 reports; 75 with critical findings (test set)	Sensitivity 0.92; Specificity 0.99	High specificity reduces false-positive audit burden; feasible for communication quality oversight
Dattu et al. [15]	RF, ANN, SVM	Predictive maintenance of critical medical devices	2,800 maintenance records; 6 hospitals; 10 device categories	RF/ANN accuracy 1.00, F1 0.90; SVM recall 0.94; estimated 12–15% improvement in spare-parts availability	Reduces equipment downtime; supports operational quality and patient safety through reliable device availability
Yun et al. [16]	Deep learning (CNN)	Mammography phantom image QA (national accreditation program)	5,813 phantom images (after preprocessing)	Accuracy: fibers 87.84%, specks 93.43%, masses 86.63%; AUC > 0.97 for lesion presence, > 0.94 for abnormality detection	Improves consistency and efficiency of accreditation-linked imaging QC; reduces inter-reader variability

**Table 3:** AI governance and implementation requirements: evidence from included studies

Governance Domain	Key Requirement Identified	Supporting Studies	Hospital Quality Implication
Local validation	Local acceptance testing beyond vendor validation or regulatory approval	Lundström et al. [3], Larson et al. [4], Zanca et al. [5]	Hospitals must establish in-house validation protocols before deploying AI in quality-sensitive workflows
Performance monitoring	Continuous post-deployment monitoring; detection of drift or performance decay	Mercolli et al. [6], Larson et al. [4]	Performance monitoring must be embedded in quality management systems, not conducted as a one-off exercise
Governance structure	Documented governance frameworks, model inventories, intended-use specifications	Ranjbar et al. [1], Zanca et al. [5], Nehme et al. [20]	AI governance should be formalized within hospital quality management structure with assigned accountability
Workforce readiness	Training, role clarity, and interdisciplinary accountability among clinical, technical, and quality staff	Ranjbar et al. [1], Welzel et al. [7], McMahon et al. [23]	Quality teams must be equipped to commission, oversee, and challenge AI tools, not just consume outputs
Accreditation alignment	AI deployment linked to accreditation-specific QA requirements and inspection readiness	Larson et al. [4], Yun et al. [16], Lundström et al. [3]	Accreditation preparation should proactively incorporate AI governance documentation and evidence of local validation
Regulatory compliance	Conformity to national and international AI regulatory frameworks across the AI lifecycle	Zanca et al. [5], Zinchenko et al. [21], Nehme et al. [20]	Procurement and deployment must address regulatory classification, post-market obligations, and lifecycle documentation
Patient safety guardrails	Structured constraints to prevent hallucination, bias, and inappropriate AI decision propagation	Hakim et al. [19], Small et al. [13], Kobritz et al. [12]	Patient safety programs must include AI-specific risk assessment, escalation pathways, and confabulation mitigation plans

Discussion

This systematic review brings together 23 studies spanning AI and ML applications in hospital quality management, patient safety, and accreditation readiness. Three principal findings stand out from the combined evidence.

The strongest and most empirically grounded evidence favors AI deployment in structured, repetitive, and auditable quality workflows. Radiology communication auditing, accreditation-linked imaging quality control, and predictive maintenance of critical equipment each exhibited strong AI performance characteristics [11,15-16]. These tasks share well-defined digital inputs, stable operational workflows, and measurable outcomes, characteristics that are consistently associated with better AI performance across the broader literature. This pattern suggests that hospital quality teams considering AI adoption would do well to prioritize similarly well-bounded use cases during the initial deployment phase.

The evidence also consistently supports a model of AI-assisted quality governance rather than autonomous quality management. In surgical complication surveillance, NLP broadened case identification but did not supplant clinician-led severity classification [12]. In documentation support, LLMs reduced the burden but introduced confabulation risk that mandates human verification [13]. In patient-experience monitoring, ML-based social-media analysis generated supplementary signals that still required institutional interpretation [14]. Across these use cases, the appropriate implementation model is one of human-AI collaboration, with AI handling high-volume pattern-recognition tasks and humans retaining authority over interpretation, escalation, and quality judgment.

Local validation, continuous monitoring, and institutional governance readiness emerged as universal prerequisites across the literature. Studies and frameworks consistently argued that vendor-reported performance and regulatory clearance do not guarantee safe local use [3,4]. Hospitals must establish institution-level acceptance testing, performance monitoring, and governance structures aligned with accreditation and regulatory requirements [1,5]. The emergence of dedicated AI accreditation pathways, such as the ACR Recognized Center for Healthcare-AI (ARCH-AI) program, signals that accreditation bodies are beginning to codify these expectations [4], and hospitals that develop governance infrastructure proactively will be better positioned for future accreditation cycles.

Implications for Hospital Quality Systems

For hospital quality managers and patient safety officers, this review supports a phased, governance-first approach to AI adoption. Initial AI investments ought to target tasks that are data-rich, operationally well-defined, and directly measurable, such as critical findings auditing, equipment maintenance prediction, and imaging quality-control scoring. For higher-complexity quality functions involving clinical judgment, heterogeneous data, or high-stakes decisions, AI should be positioned as a decision-support layer with mandatory human oversight of outputs.

Procurement processes must require local acceptance testing, documented intended-use specifications, and post-deployment performance monitoring plans as standard contractual terms rather than optional considerations. Critically, hospital AI governance structures should be developed in parallel with AI procurement, not retrospectively after problems emerge. The International Organization for Standardization (ISO) 42001 AI management framework provides one practical scaffold for this institutional readiness work, consistent with international guidance on AI ethics and governance in health systems [27]. Effective AI deployment in clinical settings is contingent on data management architectures that balance governance, operational flexibility, and scalability, with hybrid models requiring further development to reduce implementation complexity and improve integration with clinical standards.

Strengths and Limitations

A notable strength of this review is its focus on hospital quality management, patient safety, and accreditation readiness as institutional domains distinct from general clinical AI. The review was prospectively registered, used blinded duplicate screening, and adhered to PRISMA 2020 reporting standards. The breadth of included evidence, spanning empirical performance studies, implementation analyses, governance frameworks, and regulatory reviews, provides a comprehensive picture of the current landscape.

The principal limitation is the heterogeneity of included studies in design, AI modality, quality domain, and outcome definition. Many included papers were implementation-oriented, governance-oriented, or conceptual rather than comparative effectiveness studies. A further limitation is that several included governance and conceptual studies were indirectly related to hospital accreditation or quality management, which may reduce the specificity of conclusions.

The majority of empirical studies were single-center or context-specific, which limits generalizability to other health systems. These constraints necessitated a primarily narrative synthesis and precluded formal meta-analytic pooling. Full risk-of-bias assessments (PROBAST, ROBINS-I, AMSTAR-2) are provided in the Appendices. The restriction to English-language publications may have excluded relevant evidence from non-English-speaking settings with active AI quality programs.

Future Research Priorities

Future research should prioritize multicenter prospective evaluations of AI tools in hospital surveillance, accreditation-linked quality control, and clinical documentation, alongside implementation science studies examining the governance structures, validation processes, and accreditation frameworks that enable or impede effective AI deployment. The field would further benefit from standardized outcome-reporting frameworks to improve comparability across hospital AI quality studies, rigorous health economic evaluations of AI-assisted quality-management approaches, an area currently absent from the literature, and equity-focused analyses examining whether these tools perform comparably across diverse patient populations, hospital types, and socioeconomic contexts.

## Conclusions

AI and ML are increasingly relevant to hospital quality management, patient safety, and accreditation readiness. The current evidence base supports their application most robustly in repetitive, digitally captured, and operationally well-defined quality tasks. Current literature consistently indicates that these technologies function most effectively as augmentative tools, enhancing surveillance, standardization, and quality control workflows with human oversight for interpretation, escalation, and accountability.

The effectiveness of AI within hospital quality systems depends not only on algorithmic performance but equally on local validation, continuous performance monitoring, governance maturity, and broader institutional readiness. Future progress in this field will depend on the integration of AI within structured quality systems, with equal emphasis on implementation science, regulatory oversight, and workflow integration. These findings support a phased, governance-driven adoption of AI and ML in hospital quality systems, rather than their use as autonomous decision-making tools.

## Appendices

Appendix 1: Literature search details

**Table 4:** Literature search strategy and details

Component	Details
Date of search	26 January 2026
Institution	AIIMS Nagpur
Databases	PubMed, Embase, and Scopus
Language	English
Timeframe	01 January 2016 to 01 January 2026
PubMed search strategy	("Artificial Intelligence"[Mesh] OR "Machine Learning"[Mesh] OR "Deep Learning"[Mesh] OR "Natural Language Processing"[Mesh] OR artificial intelligence[tiab] OR machine learning[tiab] OR deep learning[tiab] OR neural network*[tiab] OR NLP[tiab] OR large language model*[tiab] OR ChatGPT[tiab]) AND ("Quality Improvement"[Mesh] OR "Quality Assurance, Health Care"[Mesh] OR "Patient Safety"[Mesh] OR "Health Care Quality Indicators"[Mesh] OR hospital quality[tiab] OR quality management[tiab] OR quality improvement[tiab] OR clinical audit*[tiab] OR patient safety[tiab] OR healthcare quality[tiab]) AND ("Accreditation"[Mesh] OR accreditation[tiab] OR hospital accreditation[tiab] OR quality standard*[tiab] OR certification[tiab] OR JCI[tiab] OR NABH[tiab] OR ISO[tiab] OR "Joint Commission"[tiab])
Embase search strategy	('artificial intelligence'/exp OR 'machine learning'/exp OR 'deep learning'/exp OR 'neural network*' OR 'natural language processing' OR NLP OR 'computer vision' OR 'expert system*' OR 'large language model*' OR 'generative ai') AND ('quality improvement'/exp OR 'patient safety'/exp OR quality management OR hospital quality OR patient safety OR adverse event* OR incident report* OR near miss* OR quality indicator* OR audit* OR compliance OR 'risk management' OR 'root cause analysis' OR FMEA) AND (accreditation OR 'joint commission' OR JCI OR NABH OR ISO OR regulatory OR standards) AND (hospital* OR inpatient)
Scopus search strategy	TITLE-ABS-KEY ("artificial intelligence" OR "machine learning" OR "deep learning" OR "neural network*" OR "natural language processing" OR NLP OR "computer vision" OR "expert system*" OR "large language model*" OR "generative AI") AND TITLE-ABS-KEY ("quality management" OR "quality improvement" OR "patient safety" OR "adverse event*" OR "incident report*" OR "near miss*" OR "quality indicator*" OR audit* OR compliance OR "risk management" OR "root cause analysis" OR FMEA) AND TITLE-ABS-KEY (accreditation OR "Joint Commission" OR JCI OR NABH OR ISO OR regulatory OR standards) AND TITLE-ABS-KEY (hospital* OR inpatient)

Appendix 2: Risk of Bias assessment. Part A: PROBAST assessment (n = 3)

**Table 5:** PROBAST (Prediction model Risk Of Bias Assessment Tool) assessment of prediction model studies QA, quality assurance; QM, quality management; RoB, Risk of Bias Ratings: low = low risk of bias (favourable), unclear = unclear risk of bias, high = high risk of bias (unfavourable) PROBAST domains: 1 = participants, 2 = predictors, 3 = outcome, 4 = analysis. Ratings: low risk of bias (favorable), unclear, high risk of bias (unfavorable).

Study (Year)	Domain 1 Participants	Domain 2 Predictors	Domain 3 Outcome	Domain 4 Analysis	Overall RoB	Applicability to Hospital QM
Heilbrun et al., 2019 [11]	Low	Low	Low	Low	Low	HIGH Single-center retrospective, radiology QA workflow directly applicable
Yun et al., 2025 [16]	Low	Low	Low	Low	Low	HIGH National accreditation program data, directly applicable to imaging QC
Dattu et al., 2025 [15]	Low	Unclear	Low	High	High	HIGH Multi-site; applicable to biomedical-engineering QM, A reported classification accuracy of 1.00 with no external validation set is incompatible with realistic noise levels

Appendix 3. Risk of Bias assessment. Part B: ROBINS-I assessment (n = 4)

**Table 6:** Risk of bias assessment of included observational and implementation studies using the ROBINS-I (Risk Of Bias In Non-randomized Studies of Interventions) tool Ratings: low = low risk of bias; moderate = moderate risk of bias; high = high risk of bias; serious = serious risk of bias. ROBINS-I domains assessed: confounding, selection bias, classification, deviations from intervention, missing data, measurement, reporting bias. Ratings: low, moderate, high risk of bias. *Rahim et al. was the only study rated SERIOUS overall. It was observed to have serious selection bias as only hospitals that had active Facebook review sections were included, and reviews were written voluntarily by a self-selected fraction of patients in only two languages.

Study, Year	Confounding	Selection	Classification	Deviations	Missing Data	Measurement	Reporting	Overall RoB
Kobritz et al., 2023 [12]	Moderate	Low	Low	Low	Unclear	Low	Low	Moderate
Small et al., 2025 [13]	Moderate	Moderate	Low	Low	Low	Moderate	Low	Moderate
Rahim et al., 2021 [14]	Moderate	Serious	Moderate	Low	Moderate	Moderate	Low	Serious*
Sunami et al., 2024 [17]	Moderate	Moderate	Low	Low	Low	Low	Low	Moderate

Appendix 4: Risk of Bias assessment. Part C: AMSTAR-2 assessment (included systematic review (n = 1))

**Table 7:** Quality assessment of the included systematic review using AMSTAR-2 (A MeaSurement Tool to Assess systematic Reviews) Reference: [2]

Item No.	AMSTAR-2 Item	Rating	Rationale
1*	Did the research questions and inclusion criteria for the review include the components of PICO?	Yes	Protocol registered on OSF with defined PICO
2*	Did the report contain an explicit statement that methods were established prior to the review, and did the report justify significant deviations from the protocol?	Yes	Protocol pre-registered on OSF. Conducted per PRISMA guidelines. No significant deviations noted.
3	Did the review authors explain their selection of study designs for inclusion?	Yes	Inclusion limited to original research evaluating LLM accuracy on healthcare examinations; exclusion of commentaries, editorials, and reviews explicitly stated.
4*	Did the review authors use a comprehensive literature search strategy?	Yes	Searched five databases with comprehensive MeSH terms and Boolean combinations
5	Did the review authors perform study selection in duplicate?	Yes	Two independent screeners with discrepancies resolved by a third author
6	Did the review authors perform data extraction in duplicate?	Yes	Two reviewers independently extracted data on Covidence; discrepancies resolved by third author
7*	Did the review authors provide a list of excluded studies and justify the exclusions?	Partial yes	PRISMA flow diagram provides category-level exclusion counts. Individual excluded studies not listed.
8	Did the review authors describe the included studies in adequate detail?	Yes	Table 1 and the Appendices detail each substudy.
9*	Did the review authors use a satisfactory technique for assessing risk of bias in individual studies?	Yes	Two independent assessors with third-party adjudication.
10	Did the review authors report on the sources of funding for the studies included in the review?	No	Funding sources of individual included studies were not reported or discussed.
11*	If meta-analysis was performed, did the review authors use appropriate methods for statistical combination of results?	Yes	Random-effects inverse-variance model.
12	If meta-analysis was performed, did the review authors assess the potential impact of risk of bias on the results of the meta-analysis?	No	No sensitivity or subgroup analysis stratified by risk-of-bias ratings.
13*	Did the review authors account for risk of bias when interpreting/discussing the results?	Partial yes	Discussion acknowledges 31.3% high-bias and 46.9% some-concerns studies and notes concerns about reliability.
14	Did the review authors provide a satisfactory explanation for, and discussion of, any heterogeneity observed in the results?	Partial yes	I² of 100% reported across all analyses.
15*	If quantitative synthesis was performed, did the review authors carry out an adequate investigation of publication bias and discuss its likely impact?	No	No funnel plot, Egger test, or other publication bias assessment reported.
16	Did the review authors report any potential sources of conflict of interest, including any funding received for conducting the review?	Yes	Conflicts of interest section present: funding disclosed
Overall AMSTAR-2 confidence rating		Low	Critical weaknesses in two critical domains: (1) publication bias not assessed (Item 15), and (2) impact of risk of bias on pooled estimates not formally modelled (Item 12). Results should be interpreted with caution.
